# Statistical model choice including variable selection based on variable importance: A relevant way for biomarkers selection to predict meat tenderness

**DOI:** 10.1038/s41598-019-46202-y

**Published:** 2019-07-10

**Authors:** M. P. Ellies-Oury, M. Chavent, A. Conanec, M. Bonnet, B. Picard, J. Saracco

**Affiliations:** 1 Université Clermont Auvergne, INRA, VetAgro Sup, UMR Herbivores, F-63122 Saint-Genès-Champanelle, France; 2 INRIA Bordeaux Sud-Ouest, CQFD Team, F-33400 Talence, France; 3 Université de Bordeaux, IMB, UMR 5251, F-33400 Talence, France; 4 ENSC - Bordeaux INP, IMB, UMR 5251, F-33400 Talence, France

**Keywords:** Computational biology and bioinformatics, Structural biology

## Abstract

In this paper, we describe a new computational methodology to select the best regression model to predict a numerical variable of interest *Y* and to select simultaneously the most interesting numerical explanatory variables strongly linked to *Y*. Three regression models (parametric, semi-parametric and non-parametric) are considered and estimated by multiple linear regression, sliced inverse regression and random forests. Both the variables selection and the model choice are computational. A measure of importance based on random perturbations is calculated for each covariate. The variables above a threshold are selected. Then a learning/test samples approach is used to estimate the Mean Square Error and to determine which model (including variable selection) is the most accurate. The R package modvarsel (MODel and VARiable SELection) implements this computational approach and applies to any regression datasets. After checking the good behavior of the methodology on simulated data, the R package is used to select the proteins predictive of meat tenderness among a pool of 21 candidate proteins assayed in *semitendinosus* muscle from 71 young bulls. The biomarkers were selected by linear regression (the best regression model) to predict meat tenderness. These biomarkers, we confirm the predominant role of heat shock proteins and metabolic ones.

## Introduction

In statistical modeling, it is crucial to select the best model to accurately predict a variable of interest *Y* with a *p*-dimensional vector of covariates *X* = (*X*_1_, …, *X*_*j*_, …, *X*_*p*_). Moreover, whatever the type of model (parametric, semi-parametric or non parametric), it is also necessary to select the most interesting explanatory variables strongly linked to *Y*. Usually the procedure of variables selection is specific to the statistical method used to estimate the chosen model. Stepwise regression or lasso regression for instance select covariate in a parametric linear regression model. In this paper, we propose a new computational methodology that simultaneously selects the best regression model and the most interesting covariates. A major advantage is that this methodology is universal/generic in the sense that it can be applied whatever the type of regression model/method. Moreover, a second advantage is that the proposed approach does not rely on strong probabilistic hypotheses (such as distribution of the error term). Usually, each regression model/method has their own variable selection and evaluation procedures which can be technically/theoretically difficult to handle. In addition, they do not allow to compare performances of various regression models/methods in competition and then to retain the most relevant one. The procedure of variable selection performs a measure of importance for each covariate *X*_*j*_ by estimating the response variable with random perturbations of *X*_*j*_. The variables above a cutoff value (defined for instance via a change point criterion) are selected. Then different regression models (including variable selection) are compared using a learning/test samples approach to estimate the Mean Square Error (MSE). In practice, this methodology is likely to be applied to any regression datasets with the R package **modvarsel** (MODel and VARiable SELection) which implements this computational approach. After checking the good behavior of the methodology on simulated data, the R package is used to select the proteins predictive of tenderness among a pool of 21 potential proteins assayed in *semitendinosus* muscle from 71 young bulls.

## Three Different Regression Models

In this paper, three regression models (parametric, semiparametric and nonparametric) are considered and estimated respectively by multiple linear regression (MLR), sliced inverse regression (SIR) and random forests (RF).

In parametric regression, the underlying link function between *Y* (the response variable) and *X* (the *p*-dimensional covariate) relies on a finite number of parameters to be estimated. The most popular parametric regression model is the linear regression model $Y={\beta }_{0}+\sum _{j=1}^{p}\,{\beta }_{j}{X}_{j}+\varepsilon$ where *β*_*j*_ ∈ ℝ, *j* = 0, …, *p* are the parameters to be estimated and *ε* is an random error term. Several estimation methods exist like for instance multivariate linear regression^1^, principal component regression^2^, ridge regression^3^… Whatever the method chosen to estimate the parameters *β*_*j*_, the estimated link function $\hat{f}(X)={\hat{\beta }}_{0}+\sum _{j=1}^{p}\,\widehat{{\beta }_{j}}{X}_{j}$ gives a prediction $\hat{Y}=\,\hat{f}(X)$ of the variable of interest for a given value of the covariate *X*. MLR^1^ uses ordinary least squares for estimating the unknown parameters *β*_*j*_, *j* = 1, …, *p*. The principle of least squares is as follows: minimizing the sum of the squares of the differences between the observed response variable *y*_*i*_ in the given dataset and its prediction ${\hat{y}}_{i}$. Note that no assumption on the distribution (such as normality) of *ε* is needed to have an unbiased estimator of the *β*_*j*_ parameters. The normality assumption is only necessary to make inference. The random error term *ε* is independent of *X* with a null expectation.

In nonparametric regression, the class of the link functions is expanded to have a more important flexibility. The analytic expression of the link function is not specified and the model writes *Y* = *f*(*X*) + *ε*. The link function is estimated for instance with a Random Forests^4,5^ (RF) and a prediction $\hat{Y}\,=\,\hat{f}(X)$ is made without knowing the exact shape of *f*. RF is one of the most used supervised learning algorithm that can be easily used for both classification and regression problems. The RF model can be viewed as an additive model of the following form: *Y* = *f*_0_(*X*) + *f*_1_(*X*) + *f*_2_(*X*) + … + *ε*. Predictions are obtained through an ensemble classifier combining among many decision trees. No assumption is made about the random error term *ε* except that it is assumed to be independent of *X*. Contrary to linear models, non-linear interaction between *X* and *Y* can be taken into account. Note that only when the dimension *p* is one or two, a graphical representation of $\hat{Y}$ against *X* gives an idea of the shape of the link function. It is then difficult to interpret the shape of the link function contrary to parametric regression model where the shape is chosen a priori. For instance in multiple linear regression, a story can be told like if *X*_*j*_ goes up by 1 unit then *Y* will go up by *β*_*j*_ units, etc. However, the assumption made regarding shape of the data with a parametric approach can potentially lead to estimate a model which does not reflect the true shape of the data. To resume, the problem of regression is to estimate the link function as accurately as possible while keeping this estimation as tractable and understandable as possible. In the parametric framexork, the link function belongs to a parametric family of functions and the goal is to estimate the underlying fiit dimension parameter describing the family. Contrariwise, in the nonparametric framework, very few assumptions are made about the shape of the link function. So, nonparametric models are potentially more applicable than the parametric ones. Nevertheless, this gain of flexibility has a defect. Nonparametric regression suffers from the curse of dimensionality. Its efficiency deteriorates sharply when the dimension p of the covariate X increases.

To circumvent this drawback, it is possible to combine dimension reduction and nonparametricregression via semiparametric regression model. Here we focus on semiparametric single index model where the response variable *Y* only depends on a linear combination $\sum _{j=1}^{p}\,{\beta }_{j}{X}_{j}\,$ of the covariates (called the index) through an unknown link function *f*. This model writes then $Y=f(\sum _{j=1}^{p}\,{\beta }_{j}{X}_{j})+\varepsilon$ and a two-step regression can be used to estimate the parameters *β*_*j*_ and the link function *f* (a functional parameter). Note that *ε* is independent of *X* and its distribution is arbitrary and unknown. The first step concernes the estimation of the index $\sum _{j=1}^{p}\,{\beta }_{j}{X}_{j}\,\,$ using for instance SIR^6,7^ and the second step consists in estimating the link function *f* using for example a kernel method^8^ or spline smoothing^9,10^ on the estimated index. A prediction $\hat{Y}=\hat{f}(\sum _{j=1}^{p}\,\widehat{{\beta }_{j}}{X}_{j})$ is then made for a given value of the covariate *X*. This regression model is called semiparametric, with a parametric (resp. non parametric) part via the index (the link function). The two main advantages of this semiparametric model are:

- to keep a practical interpretation easier via the index and make charts like scatterplot of Y versus the estimated index, and to measure the impact of each covariate *X*_*j*_ on *Y* based on the estimated index;
- to overcome the curve of dimensionality in the kernel estimation of *f*: thanks to the index, the dimension of the explanatory part is decreased from p to 1, and so the objective is well achieved.

## Searching for Tenderness Biomarkers

Producing high value cuts with an homogeneous quality is an ongoing challenge for the red meat industry. Moreover, it is well known that consumers have gradually less time to cook. Thus, there is a growing demand for products that are quick and easy to prepare. However, these properties are generally not well developed in fresh meat. Among meat descriptors, tenderness is one of the most important attribute, and its wide inconsistency is a major problem for beef industry^11,12^.

Tenderness can be evaluated either by objective methods by soliciting trained panels, or by subjective methods, with a panel constituted of consumers^11,13^.

Shear force is a routine instrumental measure that might be considered as a proxy for sensory tenderness. In comparison to sensory evaluation, this method is relatively inexpensive, rapid and reproducible and it is also an alternative to sensory panels. Moreover, Shackelford *et al*.^14^ already established associations between these two methods^14–16^. Nevertheless, Holman *et al*.^17^ demonstrated that the standard at which shear force protocols are described often omit key information, leading to non-reproducible results and thus to misinterpretations.

Nevertheless, these methods are greedy in time and in money but also difficult to organize. Thus, there is a need to find a way to guarantee consistent eating quality to consumer and to characterize meat quality as early as possible. Thus, the identification of meat quality biomarkers are of great interest, especially if there are quantifiable on alive animal or early *post-mortem* on the carcass. Indeed, they will allow to orientate meat production toward the most adapted processes in meat distribution circuits^18^.

Recognizing the fundamental importance of muscle proteins to meat quality attributes, there has been a growing interest on how muscle proteins and the genes regulating their expression relate to meat quality. Biomarkers were developed since the previous methods of tenderness evaluation namely sensory panels as well as shear force methods are destructive. Indeed, these methods require removing a piece of steak from the carcass to perform the measurement hence leading to carcass depreciation, time consuming and ill-suited to day-to-day decision-making for carcass orientation. Thus, some researches were focused on tenderness determinism with the aim of better explaining and better predicting this parameter, thanks to the quantification of biomarkers (genes, proteins, metabolites). The quantification of the abundance of molecules such as proteins is of interest. Indeed, it allows to understand the interaction between genetic and environmental factors that contribute to the development of meat quality^19–25^. Potential markers of meat tenderness have been screened according to the metabolic or biological process they are involved in^23,24,26,27^. Such screening has allowed the identification of different groups of functions among protein biomarkers. The three most important groups of functions are glycolytic and oxidative energy supplying pathways and Heat Shock Proteins (HSPs)^24^. Several studies have reported the differential expression of chaperone proteins, specifically small heat shock proteins (sHSP), in muscle with variable tenderness^18,28^. Moreover, Gagaoua *et al*.^29^ indicated that proteins with cell protective functions, particularly anti-oxidative proteins and HSPs seems to play key roles in tenderness determinism.

Thus, the challenge now is to select in a list of molecular biomarkers the ones that could be used to predict meat quality, a cognitive and applied objective strongly expected both by meat scientists and by the meat industry. The objective is to provide scientists tools to identify from their own list of biomarkers, a subset of few molecules to quantify as a proxy of a targeted phenotype, in this study meat tenderness.

## Method

### Description of the proposed statistical methodology

The aim of the proposed methodology is to choose among several regression methods the best one to predict a response variable *Y* with a selection of *p*′ ≤ *p* covariates strongly linked to *Y*. Whatever the regression method, the model is estimated with a sample *S* = {(*x*_*i*_, *y*_*i*_), *i* = 1, …, *n*} of *n*observations of the covariate *X* and the response variable *Y* and the predicted values are ${\hat{y}}_{i}=\hat{f}({x}_{i})$ where $\hat{f}$ is the estimated link function.

Let us first describe a general procedure to select interesting covariates in the regression model which can be used with any regression method. This procedure performs a measure of importance for each covariate *X*_*j*_ by estimating the response variable with some perturbations of the covariate and computing the error due to these perturbations. The variable importance (*VI*) of the covariate *X*_*j*_ is then

$$V{I}_{j}=\frac{1}{n}\sum _{i=1}^{n}\,{({y}_{i}-{\hat{y}}_{i}^{(j)})}^{2}$$

where ${\hat{y}}_{i}^{(j)}={\hat{f}}^{(j)}({x}_{i})$ is the predicted value when the observations of the *j*th covariate are randomly permuted in the sample *S* and ${\hat{f}}^{(j)}$ is the new estimated link function. If the covariate *X*_*j*_ has an effect on *Y*, the random permutation of its observations will affect the prediction of *Y* and increase the error measured in *VI*_*j*_. The covariates with the highest *VI* are then the most important to predict the response variable. In order to have robust estimation of the importance of the covariates, the procedure is replicated *N* times for each covariate *X*_*j*_ leading to *N* slightly different values of *VI*_*j*_: mean values and parallel boxplots can then be plotted to compare visually the importance of each covariate *X*_*j*_. It is also possible to select the covariate with a mean *V*I above a threshold. This threshold can be the original Mean Square Error (MSE) taken as baseline:

$$MSE=\frac{1}{n}\sum _{i=1}^{n}\,{({y}_{i}-{\hat{y}}_{i})}^{2}.$$

Another way to identify automatically the useful covariates is to detect a single change point position^30^ (in mean and variance) in the ordered sequence of the *p* means *VI*’s values.

Let us now describe the procedure proposed to choose the best regression method (including variable selection) to predict the variable of interest. This procedure is based on a train/test samples approach frequently used in machine learning to estimate the error of classification and used here to estimate the Mean Square Error. The idea is to randomly split the sample *S* in a train sample *S*_*train*_ (with for instance 80% of the observations) and a test sample *S*_*test*_ (with the 20% remaining observations). For each regression method, a subset of covariates is selected using the *S*_*train*_ sample. Then each model (build with the selected covariates) is estimated (trained) using the observations in *S*_*train*_. Finally the *n*_*test*_ observations in *S*_*test*_ are used to predict with this trained model the response variable *Y* and to calculate the so-called test Mean Square Error:

$$MS{E}_{test}=\frac{1}{{n}_{test}}\sum _{i\,\in \,{S}_{test}}\,{({y}_{i}-{\hat{y}}_{i})}^{2}.$$

The smaller the *MSE*_*test*_ is, the better is the estimated regression model to predict the response variable with new observations of the selected covariates. Estimating the error of prediction with observations that have not been used to estimate the model is a good way to avoid overfitting and to fairly compare different regression methods with different number of covariates.

Here again, in order to have a more robust estimation of the *MSE*_*test*_ (less dependent on the split in two subsamples), this procedure is replicated *M* times giving *M* values of *MSE*_*test*_ for each regression method (including covariates selection). Parallel boxplots of *MSE*_*test*_ (one boxplot per regression method) are used to visually select the most relevant method.

Because the covariates selected with a regression method can be slightly different at each replication, the occurrence of each covariate *X*_*j*_ in the final model is also informative to determine the most relevant ones. The plot for each regression method of the proportion of selection of each covariate (in the *M* final regression models) gives another idea of the importance of each covariate.

**Figure 1:**
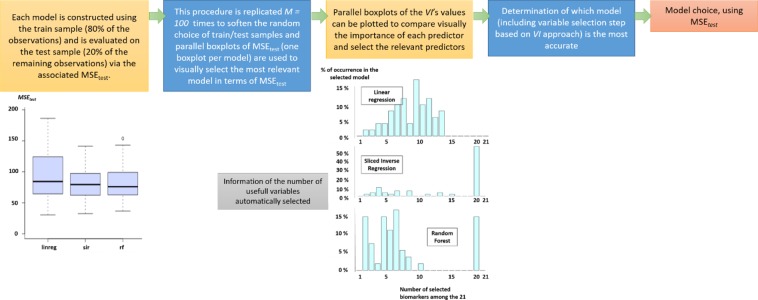
How to choose a model?

**Figure 2:**
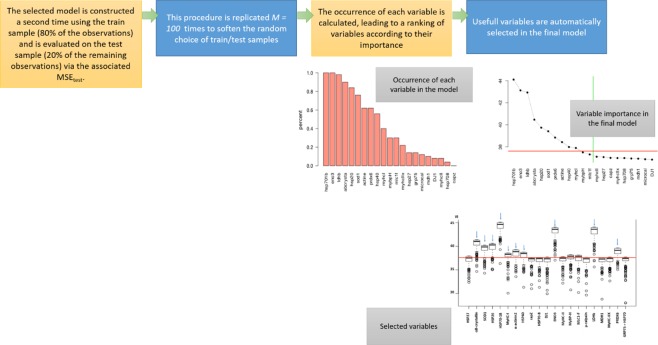
How to select variable(s)?

This methodology is briefly described in Figs 1 and 2.

### Some details about the developed R package

The R package modvarsel implements this computational methodology in two main functions:

- the function choicemod implements the train/test samples approach to determine which regression method (including or not covariates selection) is the most accurate for a given regression dataset. The following regression methods are available: MLR, SIR associated with kernel regression, RF, principal components regression, partial least squares regression and ridge regression.
- the function varimportance implements the covariate permutation technique to measure the importance of each covariate (*VI*) for any of the previous regression method.

In this paper only three regression methods are considered (MLR for linear parametric modeling, SIR for semiparametric modeling and RF for nonparametric modeling) but all the methodology is reproducible with any other regression method.

### Simulated dataset

This methodology has been first applied to simulate data generated from a fictitious model whose set of parameters have been set by the user. The general objective of a simulation study is to validate its numerical behavior.

Two regression models are considered: a parametric regression model (M1) and a semiparametric regression model (M2). Note that (M1) is linear and (M2) is non linear.

Let *X* be a *p*-dimensional variable (with *p* = 15) such that each covariate *X*_*j*_ follows a uniform distribution on [0; 0.7]. The *X*_*j*_’s are independent of each other. Let *ε* be a standard normal error, independent of *X*.

Let *β* = (4, 4, −3, −3, −2, 0, …, 0)′ be the vector of the parameters associated with each covariate *X*_*j*_.

Consequently, only the first five covariates are linked with the response variable *Y* as

$$-{\rm{M}}1:Y=X^{\prime} b+\varepsilon ,$$

$$-{\rm{M}}2:Y={(X^{\prime} b)}^{3}+\varepsilon $$

Naturally, MLR should be efficient for M1 and should suffer for M2 and SIR should be well adapted for both M1 and M2 even if the linear link function of M1 is nonparametrically estimated by kernel regression. RF is purely nonparametric and does not need to estimate the parameter *β*. However, this lack of dimension reduction can be problematic in large dimensional spaces when such a dimension reduction space exists (as in M1 and M2).

Two samples of size *n* = 200 are generated from models M1 and M2 and are used to first describe the covariate selection step and second illustrate the regression model choice.

### Experimental dataset

The methodology to compare regression methods including variables selection is also illustrated on experimental data obtained on animals coming from the EU FP6 Integrated Project ProSafeBeef (FOODCT-2006-36241). More precisely, this study was conducted using 71 young entire males of three pure breed: Aberdeen Angus (n = 21), Limousin (n = 25) and Blond d’Aquitaine (n = 25). The 12 month-old young bulls were assigned to a 100 days finishing period before slaughter and fed individually with straw (25%) and concentrates (75%). There were slaughtered at the same age (around 17 months) and final live weight (around 665 kg) in order to avoid weight and age effects on muscle characteristics and beef meat quality. All bulls were transported from the experimental farm to the experimental abattoir (slaughterhouse of INRA institute; Saint-Genès-Champanelle, France). Bulls were stunned by captive bolt prior to exsanguination, with the current ethical guidelines for animal welfare.

Samples from *Semitendinosus* muscle were excised from the carcass of each animal within 15 minutes after slaughter, frozen in liquid nitrogen and stored at −80 °C until protein extraction for protein markers quantification. The 21 biomarkers corresponded to seven biological functions^25^: ***energy metabolism***: Malate dehydrogenase MDH1, β-enolase 3 ENO3, Lactate dehydrogenase chain B LDHB; ***heat shock proteins***: αβ-crystallin CRYAB, HSP20, HSP27, HSP40, HSP70-1A/B, HSP70/Grp75 and HSP70-8; ***oxidative resistance***: superoxide dismutase DJ-1, Peroxiredoxin Prdx6, Superoxide dismutase SOD1; ***muscle fibre structure***: α-actinin 2, MLC-1F, Myosin heavy chain-I, -II and -IIx, F-actin-capping protein subunit β CAPZB, myosin binding protein H MyBP-H; ***Cell death, protein binding and proteolysis***: µ-calpain. Western blot techniques^31^ were used to specificity primary antibodies against these 21 proteins in bovine muscle. Total protein extractions were performed in a denaturation extraction buffer^32^. Bradford protein assay was used to determine protein concentration. Protein extractions were stored at -20 °C. The Dot-blot technique described by Guillemin *et al*.^31^ was used to evaluate the relative abundances of proteins.

Samples for mechanical measurement were cut into steaks 24 hours after slaughter and placed in sealed plastic bags under vacuum and kept between 2–4 °C for 14 days for ageing, then frozen and stored at -20 °C until analysis. After thawing, toughness of cooked meat was further evaluated instrumentally by Warner-Bratzler shear force using INSTRON 5944 as described by Lepetit and Culioli^33^. Force at rupture during shear compression testing was expressed in N/cm².

The aim was to select among 21 muscular biomarkers of tenderness (characterized by their relative abundances)^25^ those the most predictive of the toughness of cooked m. *Semitendinosus*.

The dataset contains then the description of the 71 young bulls on 21 variables (muscular biomarkers) and on the response variable (m. *Semitendinosus* shear force) of meat tenderness.

## Results and Discussion

This section describes the results obtained with the R package modvasel for both the simulated and the real dataset. Let us recall that the aim of the methodology evaluated here is twofold:

- identify the useful covariates based using a computational measure of variable importance (VI),
- choose the best regression method including covariates selection using mean square error (MSE) criterion based on a train/test samples approach.

### Validation of the statistical methodology via the simulation study

The sample of *n* = 200 observations generated from the regression models M1 is first used (linear model with five relevant covariates). The importances of the *p* = 15 covariates are calculated (with the R function varimportance) for *N* = 500 random replications and the method of multiple linear regression (MLR). Two graphics are obtained.

**Figure 3:**
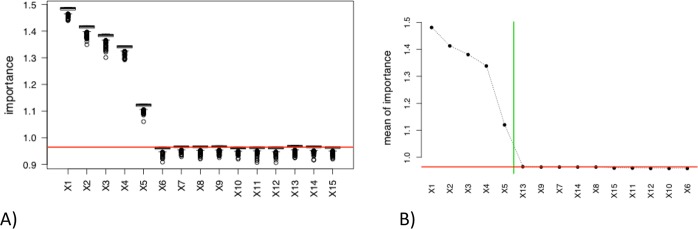
Boxplots of the 500 importance of variables (IV) values for each predictor (**A**; left) and plot of the mean of the IV’s values sorted in decreasing order (**B**; right) for model M1 and “**linreg**” estimation method. Horizontal line is the MSE value calculated on the original dataset with the “**linreg**” estimation method; Vertical line (right) is the obtained threshold with the automatic change point detection method.
- The plot of the means of these 500 VI’s values for each covariate (see Fig. 3B). In order to facilitate the graphic reading, the means are sorted in decreasing order. Vertical line is the cutoff value obtained with the automatic change point detection method. Horizontal line is again the baseline MSE.

- The boxplots of the 500 values of Variable Importante (VI) for each covariate (see Fig. 3A). Horizontal line is the MSE value calculated on the original dataset (the baseline MSE).

One can see in Fig. 3A,B that the first five covariates (associated with the largest coefficient *β*_*j*_ in absolute value in M1) have clearly greater importance than the last ten ones. The automatic covariates selection via change point detection (vertical line in Fig. 3B) works very well and keeps the five covariates relevant in the underlying model M1. The selection of the covariates with mean VI under the baseline MSE (MSE calculated on the original dataset without random permutation) selects also the five relevant variables (horizontal line in Fig. 3B). Note that the same graphics can be obtained for the other regression methods (SIR and RF for instance) but are not provided in this paper.

**Figure 4:**
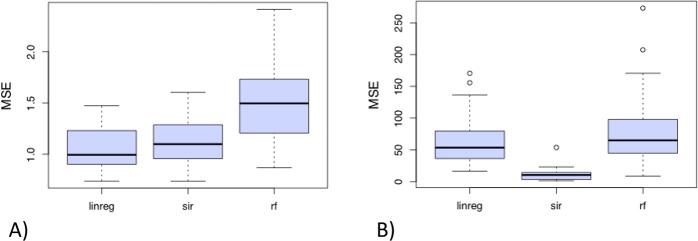
Boxplots of the N = 50 MSE’s values evaluated on the test sample when models (including variable selection) are constructed on the train sample for regression model **M1** (**A**; left) or **M2** (**B**; right) and each approach. The more the MSE is weak and the less the boxplot is displayed, better are the results.
- The barplot for each method of the number of covariates automatically selected (via change point detection) in the *N = 50* final models (see Fig. 5A,C,E). These graphics enables the user to vizualise for each regression method the complexity (measured by the number of selected variables) of the final models.

Let us now compare the performances of the three regression methods including automatic covariates selection (called **linreg**, **sir** and **rf** hereafter). For each method, the computational approach based on random train/test samples, is used to estimate the so called test Mean Square Error (*MSE*_*test*_). This procedure is repeated *N* = *50* times and three graphics are obtained:

- The boxplots for each regression method (including covariate selection) of the *N* = *50* values of *MSE*_*test*_ (see Fig. 4A). The “best” method is the one associated with the boxplot taking the smallest values of *MSE*_*test*_.

**Figure 5:**
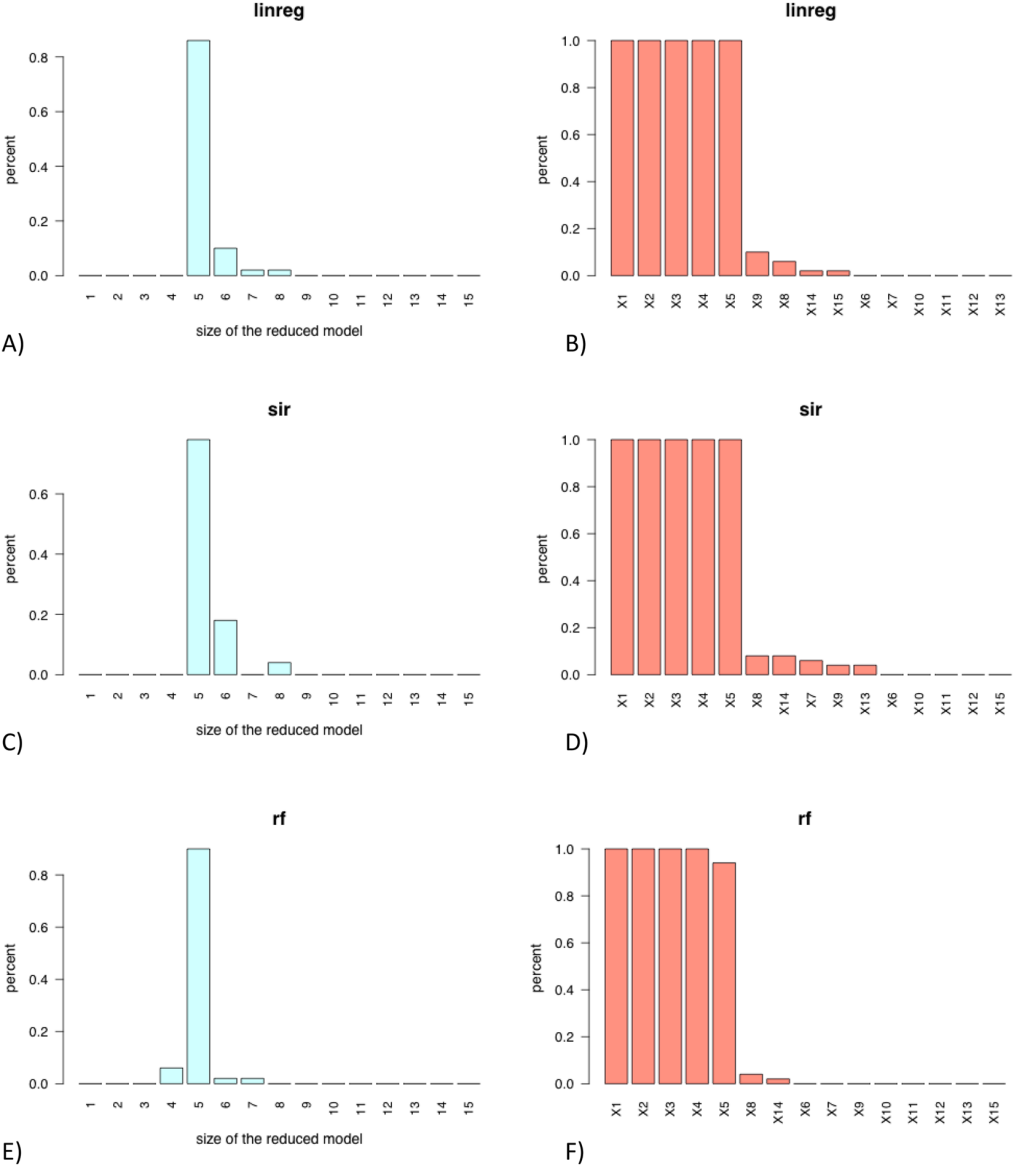
Barplot of the numbers of predictors automatically selected (via change point detection) in the final model constructed on the N = 50 train samples (left) and barplot of the occurrences of each predictor in the final model (right) for the **“linreg”** approach (**A**,**B**) for the **“sir”** approach (**C**,**D**) for the **“rf”** approach (**E**,**F**) and regression model **M1** Left: the model might be considered as stable if the percentage of a given size of the reduced model is significantly higher than the other. Right: the most often a variable is selected in the model, the most important is this variable as predictor
- The barplot for each method of the occurrences (in percent) of each covariates in the *N* = *50* final models (see Fig. 5B,D,F). These graphics are very informative to determine for each regression method the most relevant covariates.

Figure 4A shows that the methods **linreg** and **sir** are more efficient than **rf** to estimate the response variable *Y*. Their boxplots of test MSE show smaller values and lower dispersion compared to those of the **rf** method. Moreover,

- The methods **linreg** retains in 80% of cases a model with 5 covariates and a model with 6–8 covariates otherwise (see Fig. 5A). The relevant covariates *X*_1_, …, *X*_5_ are always selected in the final models (see Fig. 5B). The other variables are very rarely selected in the final models.
- The method **sir** provides a model with 5 covariates in more than 80% of cases and retains a model with 6 or 8-variables model otherwise (see Fig. 5C). The relevant covariates *X*_1_, …, *X*_5_ are again always selected in the final models and 5 other variables are sometimes selected (see Fig. 5D).
- The method **rf** shows good performances too in terms of complexity of the final models with almost 80% of 5-covariates models (see Fig. 5E). The relevant covariates *X*_1_, …, *X*_5_ are are very often selected with only variable *X*_5_ that does not appear in less than 10% of cases (see Fig. 5F). However, let us recall that Fig. 4A shows that the MSE (evaluated on the *N* test samples) of the **rf** method is significantly higher than that of the **linreg** and the **sir** methods.

To sum-up the previous results, the user may hesitate between the **linreg** and the **sir** method. Unsurprisingly (since the underlying model M1 is a linear regression model), these two approaches are the most successful in terms of MSE and in terms of selection of the five relevant covariates. As previously indicated, a parametric approach is usually preferred, as one would only have to estimate the parameters of the model, instead of having to estimate the entire model with a nonparametric approach. Moreover since a linear regression model is generally easier to manipulate, the preference for the **linreg** method may then appear more natural for the user.

What now when the underlying model is not linear? Let us consider the second sample of *n* = 200 observations generated from the regression models M2 (with five relevant covariates). This model is non linear since the link function between *Y* and the index *X*′*β* is cubic and not linear. The **linreg** method is then not well-adapted in this case while the method **sir** should easily recover the underlying structure. The **rf** method is not really sensitive to the shape of the link function but should suffer from the well-known “curse of dimensionality” since there is no dimension reduction step via an univariate index *X*′*β*. This expected result is confirmed in Fig. 4B where the methods **linreg** and **rf** are less efficient than the **sir** method to estimate the response variable *Y*. Their boxplot of test MSE show higher values and bigger dispersions compared to those of the **sir** method.

**Figure 6:**
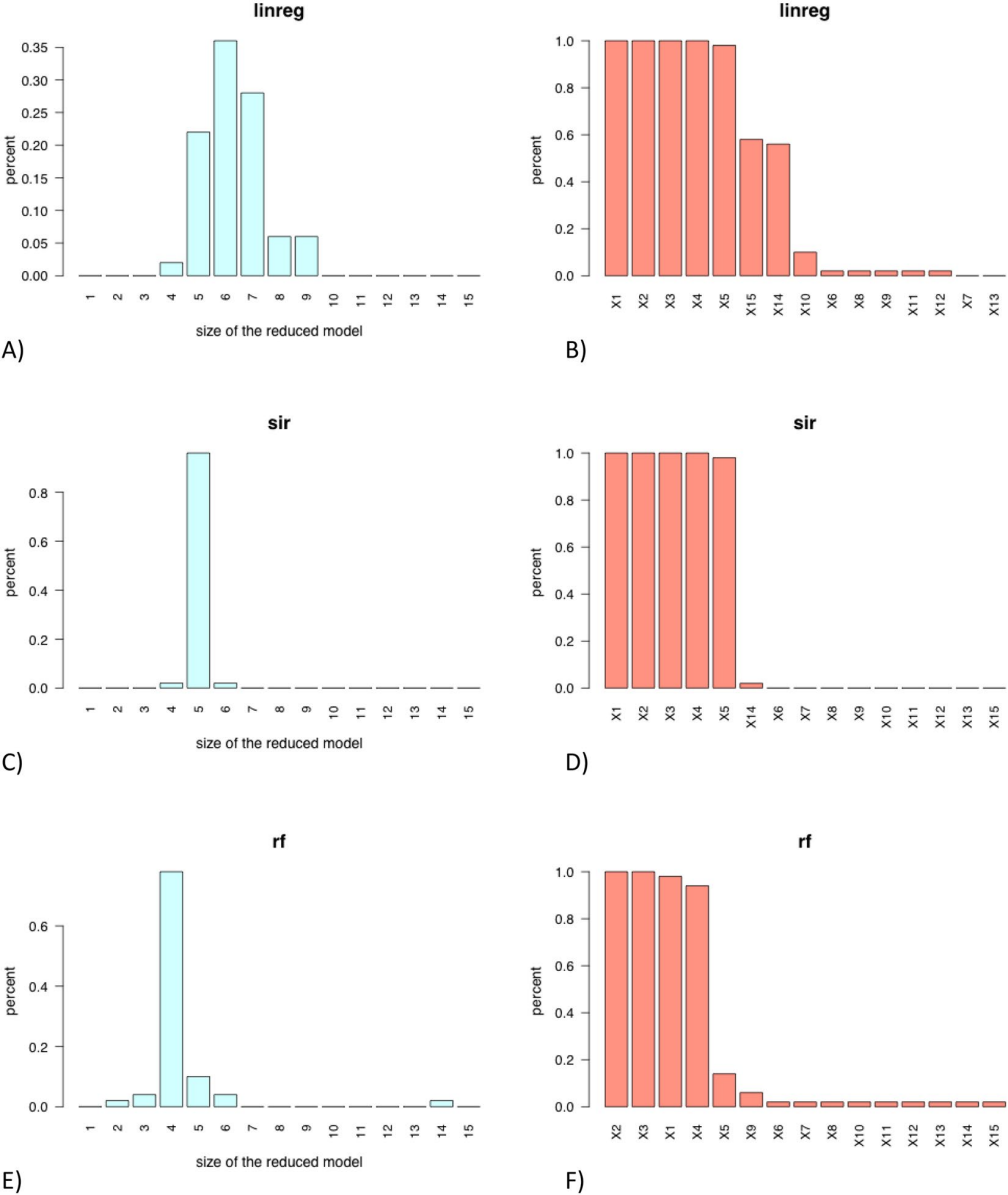
Barplot of the numbers of predictors automatically selected (via change point detection) in the final model constructed on the N = 50 train samples (left) and barplot of the occurrences of each predictor in the final model (right) for the **“linreg”** approach (**A**,**B**) for the **“sir”** approach (**C**,**D**) for the **“rf”** approach (**E**,**F**) and regression model **M2**.

The Fig. 6C shows that the **sir** method selects 5 covariates in almost 90% of cases and retains a 4 or 6-variable model otherwise. Morover the four first relevant covariates *X*_1_, …, *X*_4_ are always selected in the final models, while covariate *X*_5_ is almost always selected (Fig. 6D).

The **linreg** method selects often two many covariates. The Fig. 6A shows that 6 to 9-variables are selected in more than 70% of cases and a model with 5 covariates is only retrieved in about 20% of cases. The relevant covariates *X*_1_, …, *X*_5_ are almost always selected in the final model (see Fig. 6B) but surprisingly variables *X*_14_ and *X*_15_ are also frequently selected. The **rf** approach is less greedy in terms of size of the final models with almost 80% of models with 4 covariates (see Fig. 6E). The first four relevant covariates are again almost always selected while *X*_5_ is rarely retained in the final models (see Fig. 6F).

To sum-up the previous results, the **linreg** and **rf** methods are naturally less efficient than **sir** to predict the data simulated with model M2. Moreover the **sir** method selects often the five relevant covariates and thus retrieves the true underlying regression model. This result is not surprising since the underlying (semiparametric) regression model is a model well adapted to the **sir** approach and poorly adapted to the **linreg** approach. The **rf** approach clearly suffers from the fact that it does not build an index to reduce the dimension of the explanatory part of the model via an index of the type *X*′*β*. Therefore, for the dataset generated from M2, the preference for the **sir** approach is extremely clear for the user.

### Application to the young bulls dataset

**Figure 7:**
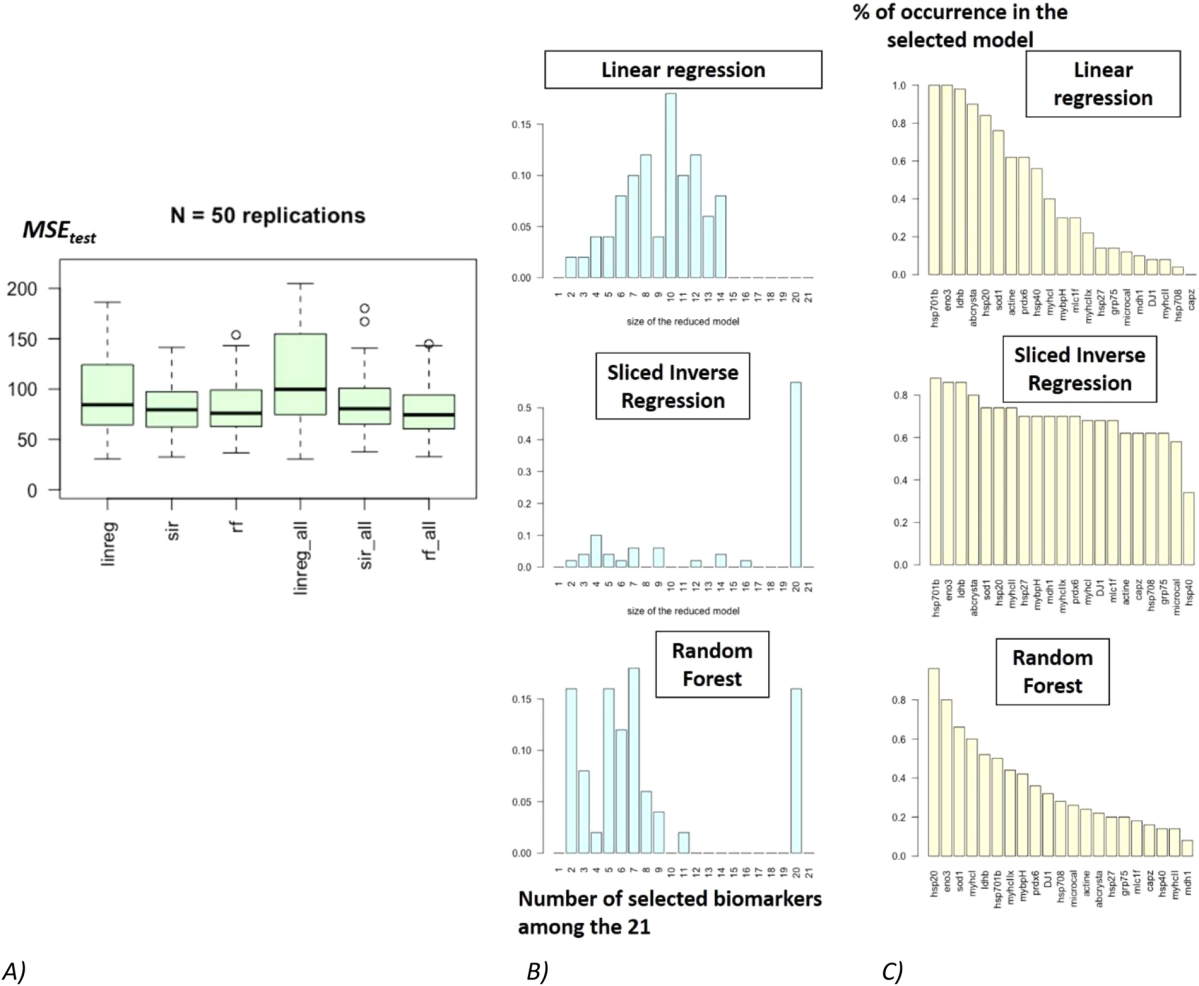
Parallel boxplots of *MSE*_*test*_ made on 100 learning/test replications (**A**); repartition (in %) of the number of selected biomarkers in each model (**B**); and percentage of occurrence of each biomarker in the selected model (**C**). (**A**) The more the MSE is weak and the less the boxplot is displayed, better are the results. (**B**) The model might be considered as stable if the percentage of a given size of the reduced model is significantly higher than the other. (**C**) The most often a variable is selected in the model, the most important is this variable as predictor. Nevertheless, if a high number of variables appears to be selected in the model, it means that the model suffers and that it is not able to select variables.

The same methodology has been applied to the dataset of 71 young bulls. However here the underlying model is unknown and the idea is to select the best model and the best covariates (relevant biomarkers) to predict the response variable (meat tenderness). The three boxplots on the left of the Fig. 7A indicates that the three methods (**linreg**, **sir** and **rf**) have very similar performances to predict the response variable. Indeed, the median test Mean Square Error of the three methods are very close with a slightly bigger dispersion when **linreg** is used. The three boxplots of the right of Fig. 7A gives the test MSE for the same three regression methods but applied with all the covariates (*i.e*. without a variable selection step). When comparing the three boxplots on the right (test MSE without variable selection step) with the three boxplot on the left (test MSE with variable selection), it appears that the selection of covariates in the final model did not deteriorate the quality of the prediction of the response variable (meat tenderness). This information is important as the main aim of the study here is also to identify a reduce number of biomarkers that could be able to predict meat tenderness (or toughness). As mentioned before, the three methods have almost identical predictive performances but the study of the biomarkers selected by each method will help to choose one of them. The Fig. 7B,C helps identifying for each method the relevant biomarkers. Figure 7B shows that the **sir** method retains a final model with 20 biomarkers (among 21) in more than 50% of cases. Moreover, almost all the biomarkers are selected in more than 60% of the final models (see Fig. 7C). The **sir** method is then not selective even if three biomarkers, namely heat shock protein 70.1B [HSP70-1b], β-enolase 3 [ENO3], lactate dehydrogenase b [LDHb] are selected in more than 80% of final models. Additionally, αB-crystallin, superoxide dismutase [c], heat shock protein 20 [HSP20] and MyHC-II [MyHCIIa + IIx] are selected in more than 70% of the final models.

With the same reasoning, Fig. 7B shows that the method **rf** retains a small number of biomarkers (between 2 and 11 biomarkers) in about 85% of final models and that 20 biomarkers are retained for the other 15% of final models. These 15% of final model with 20 biomarkers are not in favor of the choice the method **rf**. However, the progressive decrease in the percentage of selection of each biomarker in the final model (Fig. 7C) highlighted two biomarkers selected in more than 80% of the final models: HSP20 and ENO3.

Finally, regarding the ability to select few biomarkers **linreg** is clearly the best method. Figure 7B shows that with **linreg** no final model has more than 14 biomarkers and the majority is based on 10 biomarkers. The most relevant biomarkers are HSP70-1b and ENO3 selected in 100% of the cases, LDHb, αB-crystallin, HSP20 and SOD1 selected in more than 70% of the case. To sum-up, the biomarkers HSP70-1b, ENO3, LDHb, SOD1 appear to be relevant with the 3 methods, while αB-crystallin, myosin heavy chain-I [MyHCI] and HSP20 appear to be relevant with at least 2 methods.

The analysis of Fig. 7A–C leads to the conclusion the **linreg** method is, for this dataset, a reasonable choice. The predictive quality of **linreg** is very comparable to that of **sir** and **rf**, the size of the models of prediction are smaller (less biomarkers as covariates) and the linear shape of the link function is very convenient for further interpretations.

**Figure 8:**
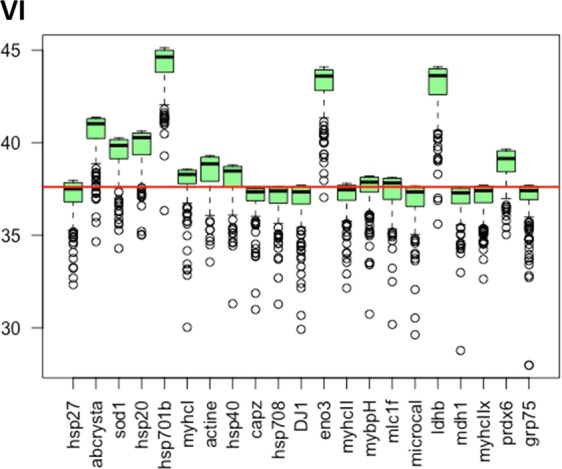
Variable importance for multiple linear regression on the entire dataset and selection of the biomarkers for the final reduced model. The variables, whose boxplots are located above the red line are sufficiently important to be considered as covariates.

Once the **linreg** method is chosen, the importance of the 20 biomarkers is now calculated (with the R function varimportance) using the entire dataset (see Fig. 8). The boxplots of the variable importance (VI) of 10 biomarkers is above the baseline MSE (horizontal line). These biomarkers are then important to predict meat tenderness and could be selected in the final model. In the present discussion, we focus our discussion only on the more important biomarkers. Finally, the 6 selected biomarkers are namely, HSP70-1b, ENO3, LDHb, SOD1, αB-crystallin and HSP20 (the 6 biomarkers that were also the most relevant in Fig. 7B).

The final model is a linear regression model with 6 covariates (the 6 selected biomarkers) used to predict meat tenderness. This model is estimated by multiple linear regression with a multiple R-squared of 0.38. The biomarkers with a positive significant link with toughness are HSP70-1B, ENO3, SOD1 and HSP20, whereas LDHb and αB-crystallin were found significantly negatively linked. This reduced list of biomarkers obtained by this computational methodology, independently to knowledge on meat biology, is however very relevant when considering the biological function of these proteins. Indeed, 3 of these proteins were heat shock proteins (HSP). This is coherent with previous results indicating an important role of HSPs in tenderization process^24,28^. Among these 3 HSPs, Hsp70-1B (selected in 100% of randomization Fig. 7C), was shown to be a biomarker of low tenderness evaluated by sensory analysis or by mechanical measurements, in different breeds and in several muscles^25^. This protein is strongly involved in the maintenance of structural, ultrastuctural and functional properties of skeletal muscle of live animals but also a role during post-mortem ageing of meat. Furthermore, an anti-apoptotic role in skeletal muscle has been described^28^. Several results showed no breed or muscle effect on the abundance of Hsp70-1B, which could explain its relation with tenderness independently of these factors. The small HSP, such as HSP20 and αB-crystallin are involved in various biological functions: preventing aggregation of partially folded polypeptides, regulating the intracellular transport and apoptotic process, regulating cellular differentiation and proliferation, translation, oxidative stress, cytoskeleton stabilization, apoptosis, and autophagy^28^. Their involvement in tenderization process has been proposed by several authors^19,28^, but their relationships with meat tenderness differ according to the contractile and metabolic properties of the muscles. In the Semitendinosus, a fast glycolytic muscle, several results showed a negative relationship of sHSP with tenderness. However, a positive correlation with the tenderness of LT muscle (mixt oxido-glycolytic) was observed^25^. In ST muscle, Guillemin *et al*.^27^ demonstrated that HSPs from both HSP70 family and small HSP family were inversely correlated with tenderness, as observed in the present study. They proposed that the tenderization efficiency could be especially dependent on the HSP20s/HSP70s ratio. These HSP families are also involved in oxidative resistance of the cell, suggesting that tenderness could be dependent on the oxidative stress^34,35^. After slaughter free radicals of oxygen (ROS) levels dramatically increase consecutively to anoxia and deprivation of oxygen explaining the important role of antioxidant enzymes. The identification of SOD1 as one of the six main protein biomarkers of tenderness in the present study, is in accordance with these data. This antioxidant enzyme protects the cell against oxidative stress which results in formation of protein aggregates that may hamper the tenderization process of the meat, thereby confirming that anti-oxidative enzymes such as SOD1 have a negative contribution to tenderness in ST muscle^27^.

Two others proteins are involved in energy metabolism, pathways strongly involved in muscle properties of living animals but also in the meat tenderization process. Indeed, ENO3 and LDHb, are involved in glycolytic metabolism, ENO3 catalyzes the conversion of 2-phosphoglycerate to phosphoenolpyruvate, and LDHb catalysis the inter-conversion of pyruvate and lactate with concomitant interconversion of NADH and NAD+. Variations of their abundance was often reported in bovine muscles differing by tenderness^25,29,36,37^. A positive correlation between LDHb abundance and tenderness of ST muscle was reported by several authors^25,37^.

## Conclusion

The originality of this paper remains in the new computational approach (which is generic whatever the considered regression models/methods) developed to choose regression model/method including variable selection. Simulations exhibited good numerical behavior of the statistical methodology. For the real dataset of 71 young bulls, whatever the regression method, the proteins relevant to predict meat tenderness are approximatively the same with close classifications by order of importance. These biomarkers are actually numbered at six: HSP70-1B, ENO3, SOD1, HSP20, LDHb and αB-crystallin. The multiple linear regression method with these six covariates has a multiple R-squared of only 0.38. Nevertheless, the biological mechanisms depend on highly regulated mechanisms remaining unknown. Moreover, it has previously been indicated that the correlations between tenderness and metabolic enzymes are different (and sometimes reversed) from one muscle to another. Thus, these conclusions needs to be confirmed on a larger and less homogeneous sampling of animals, in order to establish reliable predictions of meat tenderness. Lastly, the modvarsel R package is usable for scientist that aim to select parameters to predict a phenotype, whatever the topic.
